# Anatomical Attributes of the Optic Nerve Head in Eyes with Parafoveal Scotoma in Normal Tension Glaucoma

**DOI:** 10.1371/journal.pone.0090554

**Published:** 2014-03-03

**Authors:** Aparna Rao, Sujoy Mukherjee

**Affiliations:** 1 Glaucoma Services, LV Prasad Eye Institute, Patia, Bhubaneswar, India; 2 Diagnostic and Imaging Services, LV Prasad Eye Institute, Patia, Bhubaneswar, India; Hospital Universitario Virgen de la Arrixaca. Fundación para la Formación e Investigación Sanitarias de la Región de Murcia. Instituto Murciano de Investigaciones Biosanitarias, Spain

## Abstract

**Purpose:**

To evaluate optic nerve characteristics independent of systemic factors predisposing to parafoveal scotoma in normal tension glaucoma.

**Methods:**

We included 40 patients with bilateral normal tension glaucoma with parafoveal scotoma (visual field defect in one hemifield within 10° of fixation with at least one point at p<1% lying at the two innermost paracentral points) in only one eye (Parafoveal group, PF, n = 40) identified from the hospital database in this observational cross sectional study. The other eye with no parafoveal scotoma constituted the control group (n = 32). Red free fundus photographs were evaluated using Image J software analyzing parameters including vertical and horizontal disc diameter, disc haemorrhage, location and angular width of the retinal nerve fibre layer depth and displacement of the central vessel trunk, CVT (vertical and horizontal). Clinical characteristics and disc parameters were compared in the two groups.

**Results:**

The PF group had lower mean deviation(MD) and visual field index (VFI) and higher pattern standard deviation (PSD) than control group (p≤0.001) for similar untreated IOP, (p = 0.9). Disc haemorrhages were more frequent in the PF group, p = 0.01. The PF group had greater width of nerve fibre layer defects, p = 0.05 and greater vertical displacement of the central vessel trunk, p = 0.001. On multivariate logistic regression, parafoveal scotoma was significantly associated with increased vertical distance of the CVT, p = 0.0001.

**Conclusion:**

Increased vertical displacement of the CVT is associated with parafoveal scotoma in normal tension glaucoma. Localising the vessel trunk may help clinicians in identifying patients at risk for parafoveal involvement.

## Introduction

Glaucomatous optic nerve damage is the end point of pressure dependent and independent glaucoma. [1], [2] Factors leading to glaucomatous cupping in high tension glaucoma are well described in literature with mechanical compression and vascular factors being some postulated mechanisms for glaucomatous optic nerve damage. ^(1)^ Such descriptive mechanisms fail to explain the mechanism of glaucomatous cupping in low or normal tension glaucoma (NTG) patients where vascular factors are believed to predominate among many other risk factors [2], [3].

Several studies have tried to identify possible systemic risk factors which include vascular abnormalities like migraine, Raynaud’s phenomenon, cardiovascular insufficiency and female gender. [3] Local ocular factors like differential pressures across the optic nerve, now termed a translaminar pressure gradient and low cerebrospinal fluid pressure are now increasingly recognised as predominant risk factors for optic nerve damage at “normal” intraocular pressure (IOP). [4] Presumably, optic nerve head response to different pressure differentials across the lamina cribrosa may partly explain presence of visual field (VF) defects which are deeper and closer to fixation in NTG as compared to high pressure glaucoma or primary open angle glaucoma (POAG) eyes. A recent study demonstrated that eyes with initial parafoveal scotoma have baseline characteristics which are significantly different as compared to eyes with peripheral scotomas. [5] Studies have also reported difference in area of peripappilary atrophy among NTG eyes with IOP<15 or >15 mm Hg, suggesting IOP dependent mechanisms also actively playing a role in glaucomatous cupping. [6] Kim et al reported localized retinal nerve fiber layer defects (RNFLD) being closer to the center of the macula in the low IOP group than that in a relatively high IOP group, while no differences in angular width of RNFLD or field indices were noted between the 2 groups. [7] Anatomic differences in the optic nerve head which confer stress bearing function to different optic nerves as also to its different regions may be responsible for such observations and also late involvement of the central visual field till advanced stage of glaucoma in most eyes. Since central field is affected in advanced stages, evaluating causes of central involvement in earlier stages in some eyes with NTG would be useful. To identify these local optic nerve head parameters determining the stress response, we attempted to study differences in bilateral NTG eyes with involvement of parafoveal area in one eye only despite similar systemic risk factors (like hypertension, cardiovascular disease) influencing both eyes. This makes it easier to evaluate disc related factors responsible for early central involvement in one eye in a patient with NTG with similar systemic factors at play in both eyes.

## Methods

### Participants

Medical records of NTG patients who had undergone visual fields at our tertiary centre from 2007–2012 were reviewed and those with bilateral visual field defects were selected. Data that were retrieved included the best corrected visual acuity, family history of glaucoma, spherical equivalent, gonioscopy, slit lamp biomicroscopy, baseline untreated IOP by Goldmann applanation tonometry, central corneal thickness, dilated non-stereoscopic color optic disc photographs, Humphrey visual fields (Carl Zeiss Meditec, 24-2, 10-2 and macular program). All procedures adhered to the tenets of the Declaration of Helsinki and the study was approved by the institutional review board of LV Prasad Eye Institute. As routine protocol, a written informed consent is taken for all patients at our institute for detailed ophthalmic examination and all required diagnostic procedures. Patients with associated retinal or neurological problems with associated visual field defects were excluded.

### Below are the routine protocols followed at our institute for the following investigations-.

#### Fundus photography protocol

All images were acquired by one examiner (SM) blinded to the diagnosis or details of the patient using the Visupac version 4.4.4 (FF 450 plus IR Carl Zeiss Ltd USA). Pupils were dilated by one drop of 1% tropicamide and 2.5% phenyephrine. Only images with good quality were selected for the study while those with motion artefacts, poor contrast, clarity or inability to distinguish disc margins were excluded from the study. Photographs were taken in color and red free frames.

Those with hazy media, refractive errors ≥ −3 dioptre (D), associated posterior segment lesions like diabetic retinopathy, ARMD, vascular occlusions) or anomalous insertion or tilted disc were excluded.

#### (c) Visual field testing

**Figure 1:**
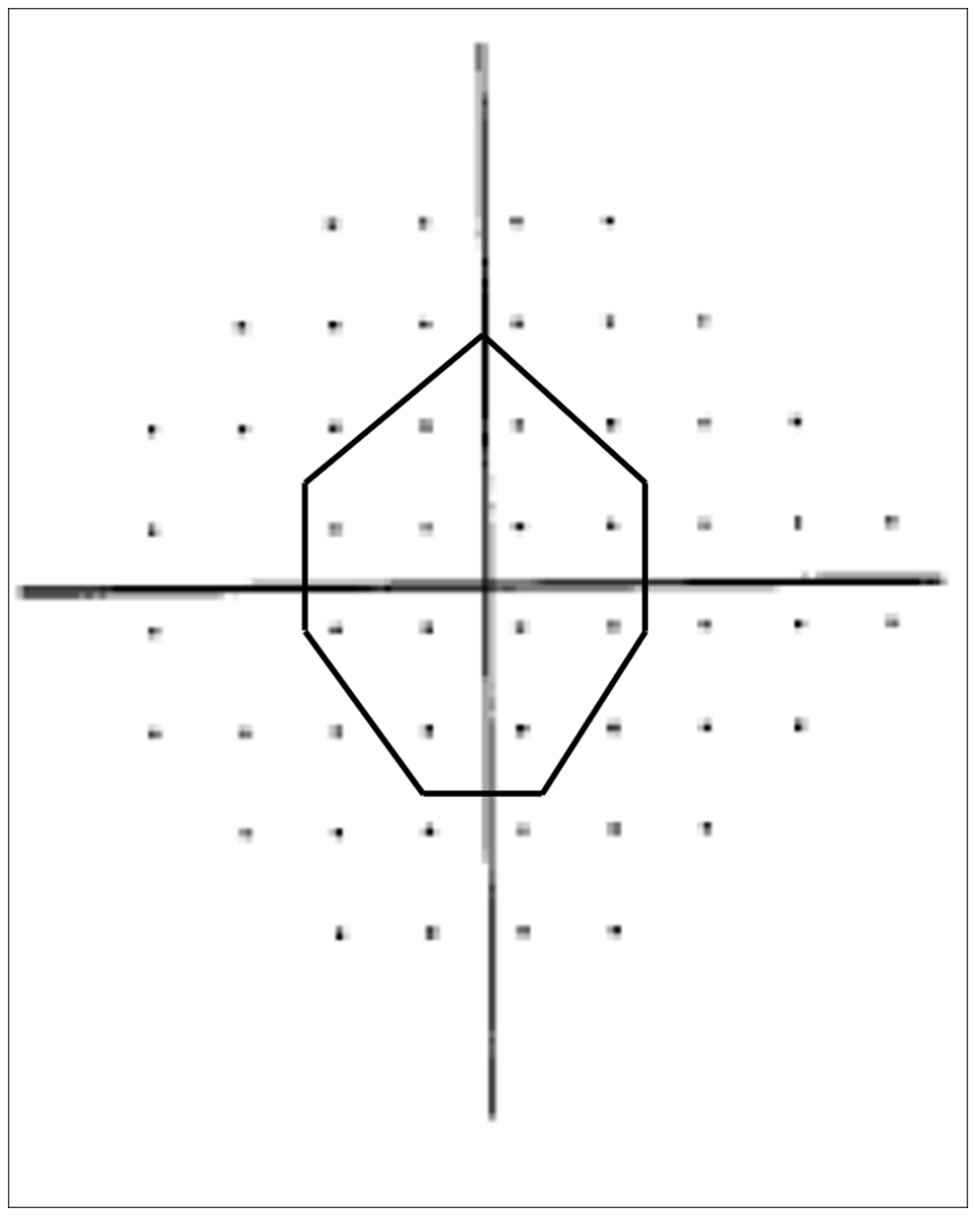
Pattern deviation plot to identify parafoveal scotoma defined by abnormal points within 12 points (shown by the dashed-line). The visual field defect would include those within 10° of fixation with at least one point at p<1% lying at the two innermost paracentral points.

Only reliable fields with defects either in the superior **or** inferior quadrant were included which included fixation losses, false-positive errors, and false-negative errors were less than 20% while those with advanced biarcuate defects were excluded. A glaucomatous visual field (VF) defect was defined by a glaucoma hemifield test outside normal limits, the presence of at least three non-edge test points in the same hemifield on the pattern deviation probability plot at p<5% with at least one point at p<1% and excluding points directly above or below the blind spot. Parafoveal (PF) scotoma was defined as a VF defect in one hemifield within 10° of fixation with at least one point at P less than 1% lying at the two innermost paracentral points, with or without defects outside the central 10° in the superior or inferior arcuate area (**Figure 1** **)**. All patients with PF scotoma in only one eye were included (eye with PF involvement henceforth termed as **PF group**). One eyed patients with PF involvement in one eye (therefore belonging to PF group) and absolute glaucoma in the other eye were also included. In all patients, the other eye with non-parafoveal defects served as control (henceforth **control group**). The visual field indices including mean deviation (MD), pattern standard deviation (PSD) and visual field index (VFI) as well as details like number of points with p<1%, defects depth on pattern deviation and foveal threshold were compared in both groups.

#### Image analysis

To avoid magnification errors, only red free images showing disc, macula and posterior pole were selected for analysis while magnified images capturing the disc alone were excluded. All images were exported to Image J software (http://rsbweb.nih.gov/ij/; www.nih.gov, National Institutes of Health, Bethesda, MD).for analysis and rescaled to a unified scale. Distances between two points of interest were measured in millimetre scale after rescaling in each of the images. All included photographs were analysed by two independent examiners (APR & SM) blinded to clinical details and an average of 3 measurements were used for analysis. Each image was then analysed using the following protocol:

#### a) Disc characteristics

**Figure 2:**
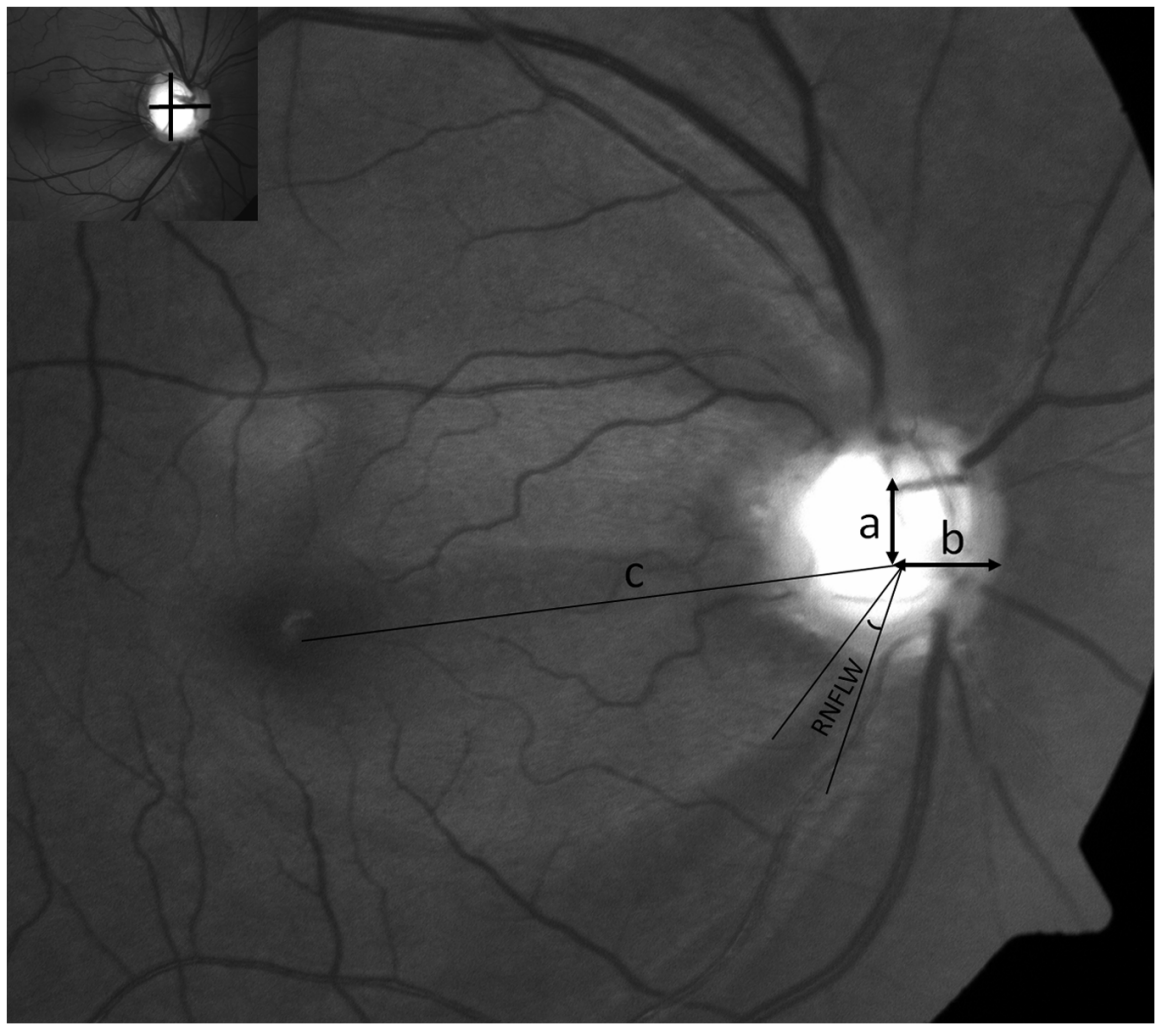
Parameters obtained from the red free fundus photograph. Reference line “c” is a line from the center of the optic disc to the center of the macula. The vertical distance of the central vessel trunk “a” and horizontal distance “b” was measured from the reference line and retinal nerve fibre layer width (RNFLW) using Image J software. Inset shows the division of the disc by two lines into 4 quadrants for determining the exit quadrant of the central vessel trunk.

The optic disc was evaluated using Image J measuring the vertical and horizontal disc diameter using the callipers function. The presence and location of disc haemorrhage was noted. The angular locations and widths of the RNFLD were measured as previously described with some modifications. [8] Briefly, a line from the centre of the optic disc to the foveal centre (the reference line), was drawn on the red free photographs. After identifying the reference line, the number of defects and the location of the defects (superotemporal, inferotemporal or PMB superior or inferior) were noted. The angular width of the defect was measured in degrees measured by the angle measurement tool in the image J software as the angle formed by the two border lines of the RNFL defect as shown, **Figure 2**. For multiple defects, the total width was calculated by summing the angular widths of all defects in that eye.

#### b) Position of Central Vessel Trunk (CVT)

The disc was divided into 4 quadrants by two lines passing through its centre into superotemporal, superonasal, superonasal and inferonasal quadrants (**Figure 2**). The exit quadrant of the CVT on the lamina cribrosa surface was then noted. The distance of the CVT was measured horizontally from the nasal disc border and vertically from the reference line by callipers in the image J software.

### Statistical Analysis

An average of 3 readings obtained by Image J software was used for final analysis. Intraclass co-efficient was used to evaluate inter-examiner reproducibility for all measurements. For assessing repeatability, 10 consecutive images were examined by each examiner and repeatability coefficients evaluated for each of the horizontal and vertical CVT distance measurements (each being measured 10 times on each image by the same examiner at the same session, Table S1). Normality was analysed using Kolmogrov-Smirnov test while descriptive data were described as means (±SD) for normally distributed and median (and range) for non-parametric variables with alpha error set at <0.05. Comparisons between the PF and control group were done using student “t” test (or Mann-Whitney) while categorical variables were evaluated using chisquare tests. Separate analysis was done to evaluate differences between eyes with untreated IOP of <15 or >15 mm Hg. Association of parafoveal involvement with independent clinical variables including age, untreated IOP, corneal thickness and optic disc characteristics as defined earlier (disc diameter, horizontal and vertical distance of CVT, width of RNFLD) and visual field indices were analysed using univariate and multivariate logistic regression. Since visual field indices including MD, PSD and VFI were observed to be collinear variables evidenced by variance inflation factor >10, they were included in three separate models. All statistical analysis was done using Stata (Stata ver. 10.0; StataCorp, College Station, TX).

## Results

We included 72 eyes of 40 patients with a mean age of 53±10.8 years (range 34–76 years). This included 8 one eyed patients (male:female = 6∶2) and 32 bilateral normal tension glaucoma (male:female = 20∶12).

The average MD and VFI in the PF group was −11±6.8 dB and 71±20.9, respectively with a mean untreated IOP of 15±2.8 mm Hg **(** **Table 1** **)**. There was no statistical difference in age (52±10.09 vs 55±11.9) or treated IOP (12±2.5 vs11±1.5), female gender (8 vs 6) or spherical equivalent (−1±0.3 vs −0.3±0.2 dioptres) between the two study groups (PF group vs control). Disc haemorrhages were more frequent in the PF group (18 of 40 eyes, 45%) than control group (6 of 32 eyes, 18.7%), p = 0.01.

**Table 1:** Characteristics of eyes with parafoveal involvement (PF group) and other eye without parafoveal involvement (control) in bilateral normal tension glaucoma.

	PF group (n = 40)	Control (n = 32)	P value
Age (years)	52±10	55±11.9	0.8
Male/Female	19∶8	7∶6	0.05
Spherical equivalent(Dioptres)	−1±0.3	−0.8±0.2	0.2
Untreated intraocular pressure(mm Hg)	15±2.8	15±1.5	0.9
Central corneal thickness(µm)	532±22.4	524±24.6	0.6
Mean deviation(dB)	−11±6.8	−7±4.6	0.001
Pattern standard deviation(dB)	10±5.5		0.001
Visual field index (%)	71±20.9	85±14.6	<0.001
Disc haemorrhages	18, 45%	6, 18.7%	0.02
Intraocular pressure <15 mm Hg	16,40%	12, 37.5%	0.6

All indices including MD, PSD and VFI were significantly better in the control group than the PF group with lower MD and VFI and higher PSD in the latter group for similar untreated IOP in the two groups. Visual field defects in the PF group included superior (n = 29) or inferior (n = 5) arcuate defects involving parafoveal area in 35 (of 40) eyes and isolated defects involving the parafoveal area in 6 eyes, **Table 2**. The control group has superior arcuate defect in 20 eyes and peripheral nasal step in12 eyes. Eyes in the PF group had a lower mean threshold of all points in the involved quadrant (superior or inferior) on the pattern deviation plot (17.2±2.4 dB) as compared to controls (25.4±4.1 dB) despite similar foveal thresholds (24 dB vs 31 dB,p = 0.06). The lowest defect depth on pattern deviation plot was also significantly worse for the PF group than control group (−31 dB vs −16 dB), p = 0.008 though the number of points with p<1% were not statistically different in the two groups, p = 0.3.

**Table 2:** Comparison of Visual field characteristics of eyes with parafoveal involvement (PF group) and control group.

	PF group (n = 40)	Control(n = 32)	P value
Superior/inferior defects	29∶5	20∶12	0.06
Mean threshold in involved quadrant (dB)	17.2±2.4	25.4±4.1	**0.04**
No of points <1%	11±7.3	−10±6.8	0.3
Foveal threshold (dB)	24±4.2	31±2.1	0.06
Visual field index (%)	71±20.9	85±14.6	
Lowest defect depth (dB)	−31±5.2	−16±6.2	**0.008**

Mean deviation correlated negatively with width of RNFLD (r = −0.4, p = 0.008) and with vertical distance of CVT (r = −0.8, p = 0.01) while PSD correlated with positively with RNFLD width (r = 0.3, p = 0.02) and vertical CVT distance (r = 0.4, p<0.001).

### Disc Measurements

The ICC for all disc measurements was good 0.91(vertical disc diameter), 0.89 (vertical CVT distance. 0.83 (horizontal CVT distance) and 0.92(width of RNFLD) while repeatability for horizontal and vertical CVT distance for the first examiner (APR) was 0.06–0.12 (Table S1).

Vertical disc diameter was greater in the PF group (2±0.8 vs 1±.0.2, p = 0.02) though horizontal disc diameter and cup disc ratio were not statistically significant, **Table 3**.

**Table 3:** Disc characteristics of eyes with parafoveal involvement (PF group) and control group.

	PF group(n = 40)	Control(n = 32)	P value
Vertical discdiameter (mm)	2±0.8	1±.0.2	**0.02**
Horizontal discdiameter (mm)	1.5±0.2	1.6±0.2	0.1
RNFL defectwidth (degrees)	63±40.02	47±39.2	0.05
Vertical CVTdistance (mm)	0.2±0.02	0.07±0.04	**0.001**
Horizontal CVTdistance(mm)	0.7±0.2	0.8±0.2	0.8
Inferior RNFLdefects	62.5%	50%	**0.03**
Superior CVTexit location	22	15	0.06

There were a mean of 1±0.6 RNFL defects per eye with 6 having papillomacular bundle defects all of which were situated in the inferior quadrant, **Figure 2**. There were more isolated RNFLD in the inferior quadrant in the PF group (25 eyes of 40 eyes, 62.5% vs 16 of 32 eyes, 50% in control group, p = 0.03) with combined defects in inferior and superior quadrant seen in the rest of eyes among both groups. The mean angular width of the RNFLD was 58±39.8 degrees (16–190°) with significantly greater width of the defects seen in the PF group (63±40.02 vs 47±39.2 degrees, p = 0.05). Comparing eyes with baseline IOP of <15 mm Hg and >15 mm Hg, we found a greater width in eyes with IOP>15 mm Hg (61±23.5 vs 54±38.4, p = 0.6). No other disc variables were found to be different among eyes with IOP< or >15 mm Hg, **Table 4**.

**Table 4:** Comparison of disc characteristics in bilateral normal tension glaucoma with low or high untreated intraocular pressure (IOP).

	IOP<15 mm Hg	IOP>15 mm Hg	P value
Vertical disc diameter (mm)	1.9±1.2	1.8±0.7	0.7
Horizontal disc diameter (mm)	1.2±0.1	1.3±0.08	0.2
RNFL defect width (degrees)	61±23.5	54±38.4	0.6
Vertical CVT distance (mm)	1±0.9	1±0.5	0.2
Horizontal CVT distance (mm)	0.7±0.2	0.6±0.2	0.6
Mean deviation(dB)	−9±3.4	−6±5.8	0.9
Pattern standard deviation (dB)	7±4.6	8±4.3	0.7

**Figure 3:**
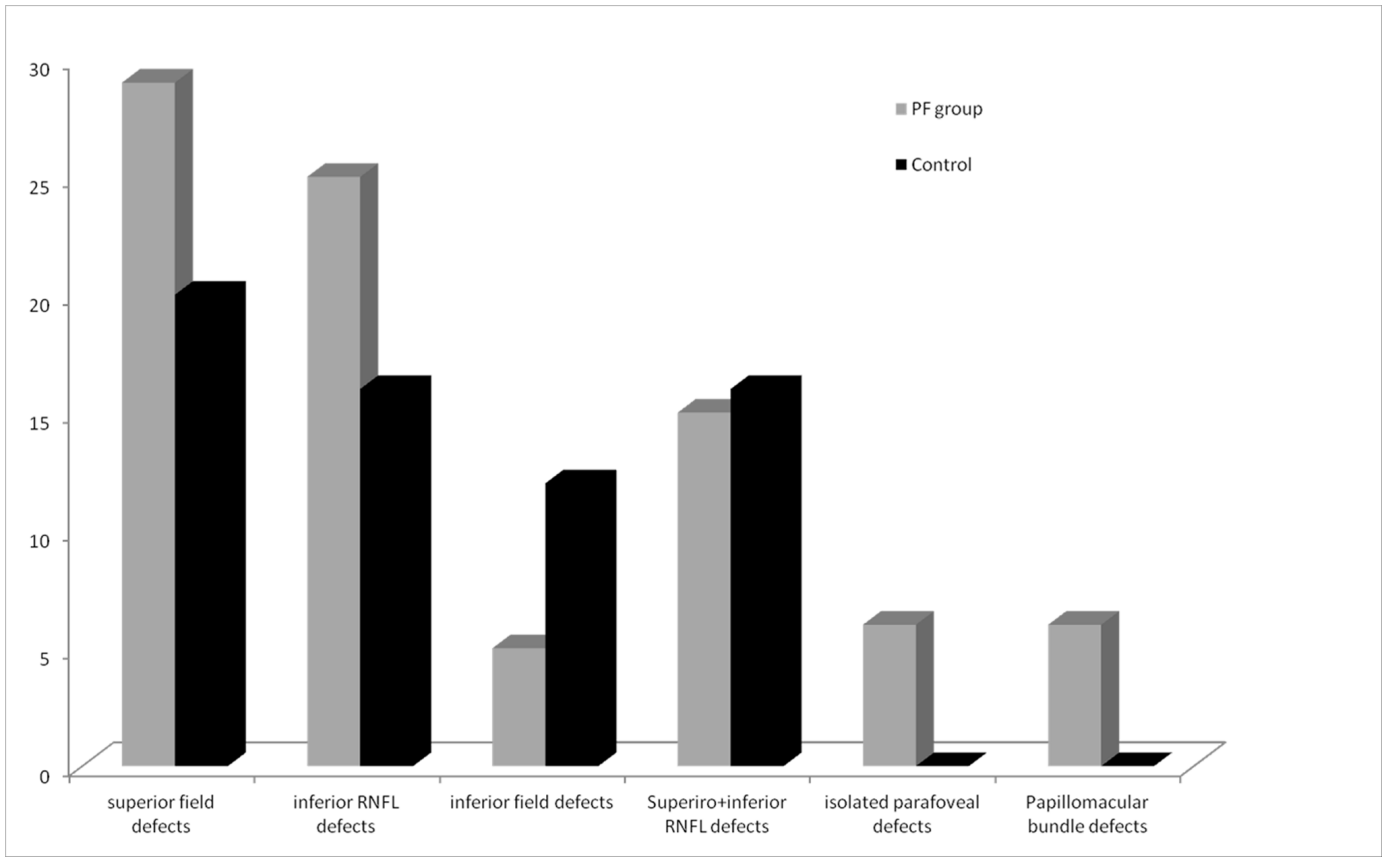
The parafoveal scotoma group showed a predominance of superior field defects (29 of 40 eyes) and inferior retinal nerve fibre layer defects (25 of 40 eyes).

**Figure 4:**
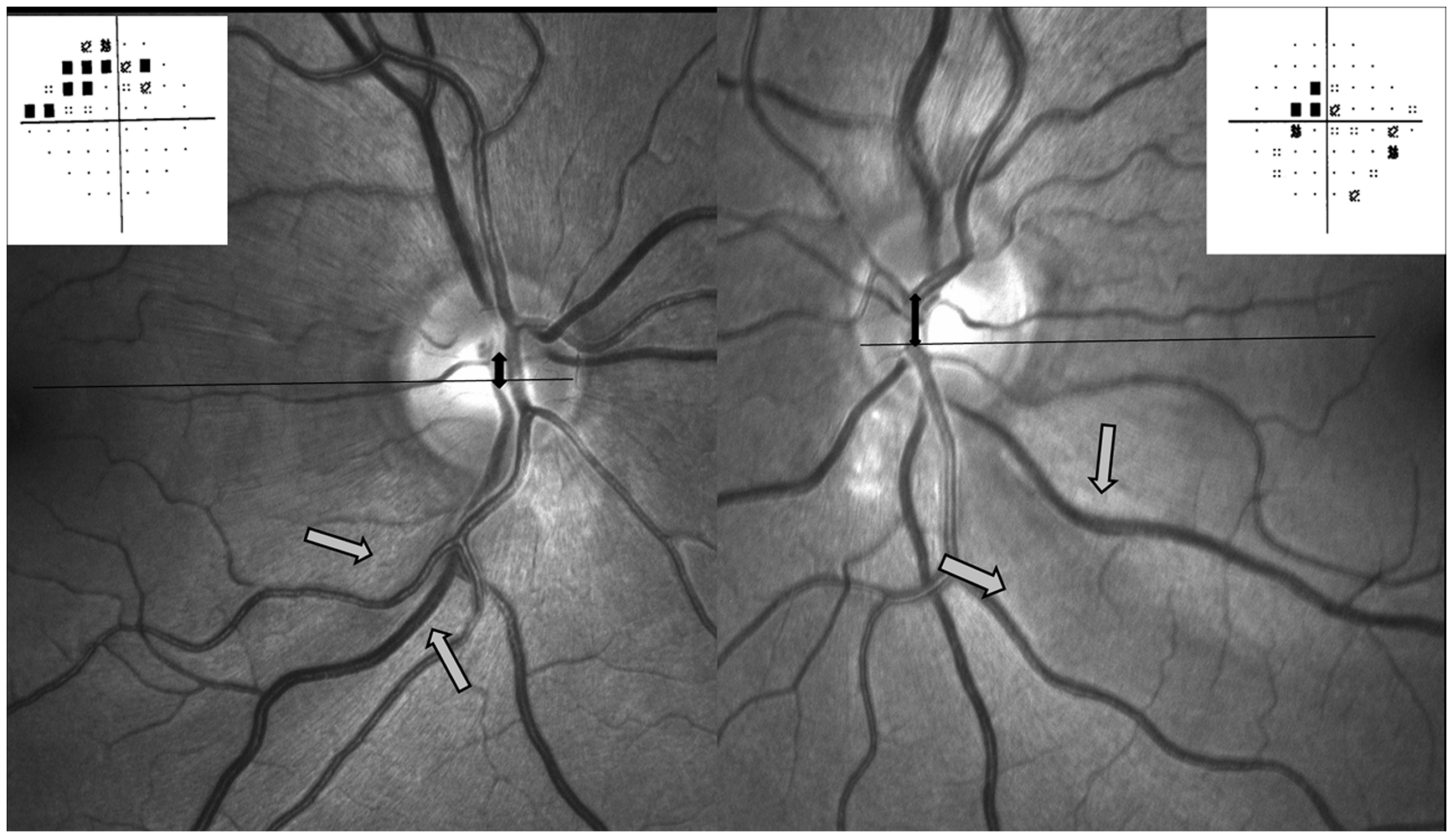
Red free Fundus photographs showing greater vertical displacement of the central vessel trunk (black solid arrows) into the superonasal quadrant away from the retinal nerve fibre layer defect inferotemporally (gray arrows) in the left eye with parafoveal scotoma (inset) as compared to the right eye with no parafoveal involvement (inset).

The CVT was situated in the superior quadrant in 43 eyes (superonasal-46, superotemporal-7) and was inferiorly placed in 29 eyes (inferotemporal-20, inferonasal-9), p = 0.02. The horizontal distance of the CVT did not differ between the two groups (0.7±0.2 mm in PF group vs 0.8±0.2 mm in control group, p = 0.8). However the vertical distance of the CVT from the reference line was found to be significantly greater in PF group (0.2±0.02 mm) than control (0.07±0.04 mm), p = 0.001, signifying a greater displacement of the CVT from the central reference line in the PF group. Eyes with inferior RNFL defects had superonasal displacement of the CVT (except 3 eyes with combined superior RNFLD having CVT exit in inferonasal quadrant). All eyes with defects in the papillomacular bundle in the inferior quadrant had superonasally placed CVT **(** **Figure 3** **)**. There was no significant difference in the CVT exit in eyes with IOP< or >15 mm Hg, **Table 4**. **Figure 4** **and** **5** shows some representative cases included in this study.

**Figure 5:**
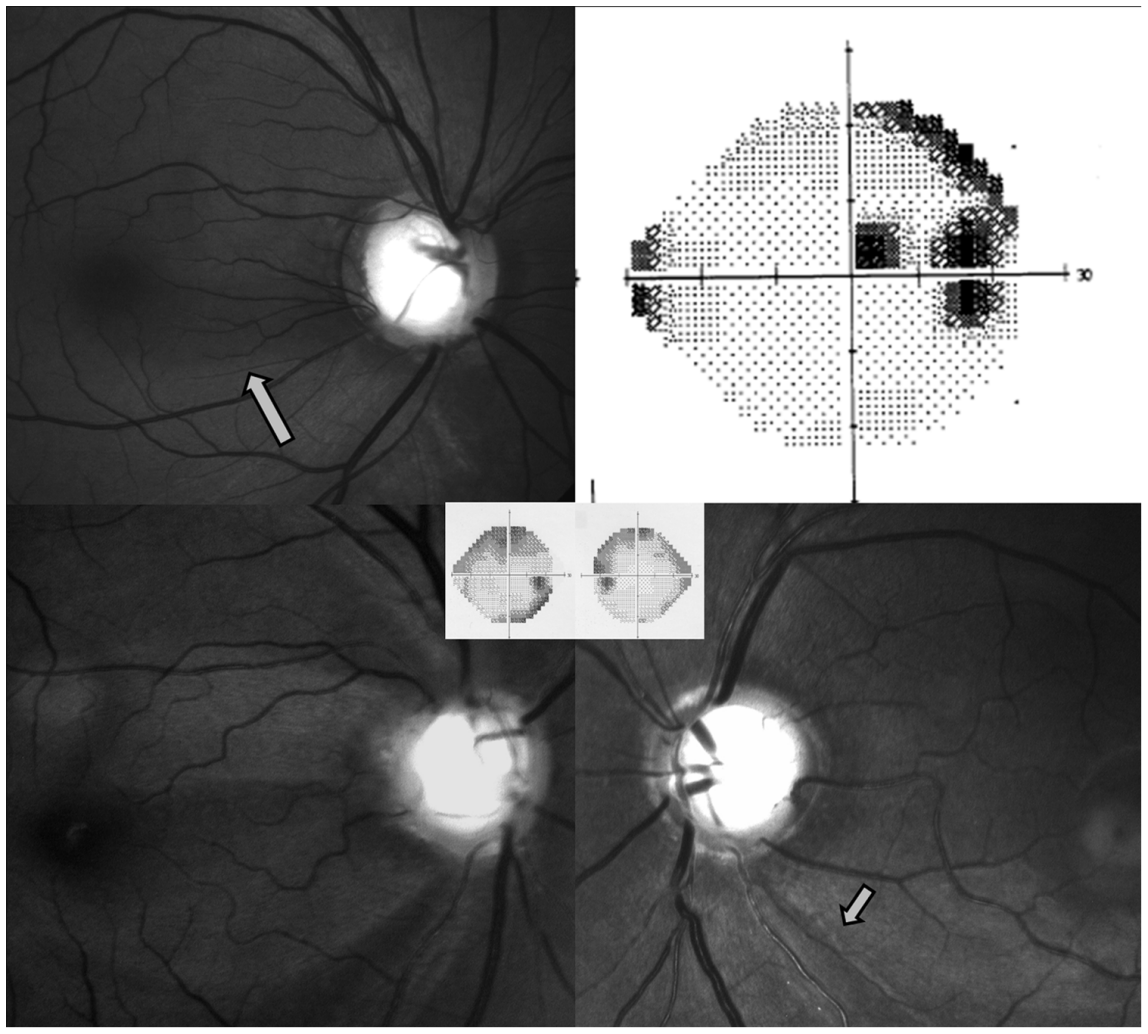
Red free photograph showing large disc in a patient with inferior papillomacular bundle defect (gray arrows) with superonasal displacement of the central vessel trunk (upper left) associated with parafoveal scotoma (upper right). Lower panel shows the red free photographs of case 6 with wedge shaped retinal nerve fibre layer defects involving superior, inferior and inferior papillomacular area, superonasally displaced central vessel trunk in the right eye and inferior defect (gray arrow) and centrally placed central vessel trunk in the left eye with parafoveal involvement in the right eye (inset).

Comparing eyes with disc haemorrhage in both groups, eyes with haemorrhages in each of PF and control group had greater vertical CVT distance (0.18±0.08 vs in PF and 0.08±0.04 in control) than those without (0.13±0.07 mm in PF group and 0.06±0.02 in control group), p = 0.06 and 0.08, respectively. The mean deviation and VFI were better in the eyes without disc haemorrhages in both groups though this was also not statistically significant in any group, **Table 5**.

**Table 5:** Comparison of disc characteristics in eyes with parafoveal involvement with or without disc haemorrhage.

	Disc haemorrhage	No disc haemorrhage	P value
Vertical disc diameter (mm)	1.7±0.4	1.6±0.7	0.8
Horizontal disc diameter (mm)	1.3±0.2	1.4±0.2	0.4
RNFL defect width (degrees)	42±30.4	39±23.6	0.3
Vertical CVT distance (mm)	0.18±0.08	0.13±0.07	0∶06
Horizontal CVT distance (mm)	0.6±0.2	0.6±0.1	0.9

On univariate regression, greater age, lower untreated IOP, increased vertical disc diameter, greater width of RNFLD, worse visual field indices and vertical distance of CVT from the reference line were significantly associated with PF scotoma. On multivariate logistic regression using MD, PSD and VFI in three different models, parafoveal scotoma was significantly associated with increased vertical distance of the CVT from the disc margin, p = 0.0001 while age, untreated IOP, disc diameter (horizontal or vertical), visual field indices including MD/PSD & VFI or width of the RNFL defects did not significantly influence the involvement of parafovel area on visual fields. We repeated the same analysis excluding 8 one eyed patients and obtained similar results with vertical distance of CVT influencing parafoveal involvement of visual fields, **Figure 4**.

## Discussion

This study observed large discs with greater vertical displacement of the central vessel trunk and worse visual field indices in eyes with parafoveal visual field defects in one eye of bilateral NTG patients. Disc haemorrhage was more frequent in eyes with PF involvement. The PF group also was observed to have greater width of RNFL defects with most the RNFLD appearing in the inferior quadrant, with corresponding displacement of the CVT to the quadrant diametrically opposite quadrant in all except 3 eyes. Since we evaluated the differences between two eyes of the same patient, influence of systemic associations like nocturnal hypotension or systemic vascular imbalance affecting both eyes were obviated and local anatomic/topographic differences in the two eyes predisposing to PF scotoma in NTG could be evaluated. Since we included eyes with involvement of superior or inferior fields, we could determine ‘disc specific” characteristics responsible for early paracentral involvement in one eye of bilateral NTG patients.

Previous investigations have demonstrated a relationship between the CVT exit on the lamina cribrosa and the extent of neuroretinal rim loss, location of visual field defects and peripapillary atrophy. [9]–[11] Neuroretinal rim loss and visual field defects follow a specific pattern related to regional susceptibilities of different disc regions. The superotemporal and inferotemporal area are the most commonly affected in early glaucoma while the nasal rim remnants may be the last to be affected in advanced glaucoma. Visual field defects follow the pattern of rim loss with the temporal island being the last to be involved in glaucoma [1], [2], [12]–[14].

This is partly explained by regional differences in lamina cribrosa with the superior and inferior regions having larger pores and higher pore-interpore tissue ratio, the lower susceptibility of thin fibres from the macula called the papillomacular bundle as compared to thicker fibres from the periphery, and also prominent backward bowing of the lamina in the superior and inferior disc regions with less connective tissue [1], [12].

Although the central field is spared till late stages of glaucoma, some eyes develop defects threatening fixation in the earlier stages of glaucoma with consequent greater risk for reduced quality of life. [5], [15], [16] Ritch and colleagues demonstrated that eyes with parafoveal defects differ significantly from those with peripheral scotomas and demonstrated a specific pattern of progression in the former group. [5] Their results supported the notion that eyes with parafoveal scotoma early in the disease process are associated with IOP independent risk factors as compared to those with peripheral scotoma. They postulated that IOP-independent factors associated with decreased or unstable ocular perfusion may preferentially damage the neuroretinal rim or RNFL closer to the papillomacular bundle in the inferior half of the optic nerve head or retina, compared with elevated IOP, though the mechanism behind the preferential involvement of the inferior part of the optic nerve head was not clear. Jung et al evaluating ocular factors like disc morphology reported narrower rim and larger cup in eyes with paracentral scotoma with thinner RNFL thickness inferiorly. [17] They postulated that these eyes may be associated with more ganglion cell death than those with peripheral scotoma despite similar level of visual field defect. Yet these studies included both eyes of the same patient which makes it difficult to segregate effect of systemic factors in central field involvement.

In our study we observed increased displacement of the CVT exit into the superonasal quadrant in eyes with parafoveal involvement in one eye of bilateral normal tension glaucoma thereby obviating the effects of systemic vascular factors. This suggests that involvement of the inferior optic nerve head, which is farthest from the central vessel trunk, is possibly due to increased distance of the inferior region from the central vessel trunk. This observation has been reported to be responsible for increased area of peripapillary atrophy and greater extent of neuroretinal rim loss in the area farthest from the location of CVT exit [10], [11].

Jonas et al has postulated that the retinal vessel trunk may act as a stabilising element which would resist a more backward bowing of the lamina and also confer greater vicinity to a richer vascular supply making it more resistant to insult by vascular dysfunction. [10] Our study results support the theory that the CVT confers a mechanical support as well as provides a source of vascular supply to areas in its vicinity in normal eyes with increased displacement away from the centre being a risk for central field involvement early in the disease process.

Disc haemorrhage was more frequent in the PF group than in control eyes in this study. This concurs with previous studies of disc haemorrhages more frequent in eyes with parafoveal involvement. [3], [5], [17] However previous studies attributed systemic hypotension, vascular abnormalities like Raynaud’s phenomenon as risk factors for disc haemorrhages. [2], [3] The reported frequency of DH detection ranges from 0% to 0.4% in normal patients, 2% to 37% in patients with primary open-angle glaucoma, 11% to 42% in patients with NTG, and 0.4% to 10% in patients with ocular hypertension. [18], [19] Disc haemorrhage was found to be a risk factor for visual field progression in NTG eyes with IOP<15 mm Hg. [20] While the debate continues if disc haemorrhage is a risk factor or sign of progression, it is proved beyond doubt that disc haemorrhages are associated with greater visual field loss and signify vascular dysfunction at the level of the optic nerve. Our study did not find any significant difference in disc variables in eyes with and without disc haemorrhages in both groups. This may support the theory that these are more related to systemic vascular abnormalities rather than the influence of local factors.

Visual field indices were worse in PF groups with the latter group having lower defect depth despite similar foveal threshold and number of points with p<1% signifying deeper and more localised visual field defects as evidenced by a greater pattern standard deviation. This is in agreement with previous studies which have demonstrated similar results. [5] Ganglion cells are mostly concentrated in the central or paracentral area with the greatest density 1 mm from the fovea, most them being midget ganglion cells. [21]–[23] The ratio of midget to parasol ganglion cells increases from 30∶1 in the centre to 3∶1 in the periphery with larger receptive field radii of the cells in the periphery. This may mean that the ganglion cells at the centre with smaller receptive field may be more susceptible to regional differences in supply of nutritional factors or blood supply than in the periphery. It may be postulated that displacement of the central vessel trunk away snaps off vascular supply to areas in its immediate vicinity; such a vascular imbalance would induce more localised damage to the ganglion cells in that specified area in NTG despite similar systemic factors say blood pressure or optic nerve blood flow or local factors like intraocular pressure. This may in part explain involvement of central area in only one eye or greater defect depth in PF group despite similar number of points with p<1% in patients with similar systemic risk factors and further studies exploring this theory would be desirable. The visual field index is more centrally weighted highlighting the importance of central visual field. The extent of displacement of the CVT exit with respect to the VFI in different areas may be interesting to evaluate in future studies.

While the role of IOP independent factors has been established earlier, several studies are focussing on IOP dependent factors in NTG eyes. [6], [7], [20] Lee et al found significantly higher mean IOP and greater IOP fluctuations in eyes with baseline untreated IOP>15 mm Hg thought the rate of progression was similar to those with untreated IOP<15 mm Hg. Wang et al reported greater area of inferior peripapillary atrophy in eyes with IOP<15 mm while Yamagami et al found IOP dependent VF damage in patients with an IOP of between 15 and 21 mm Hg. [15], [24] Similarly, RNFL defects were found to be more central in location in eyes with lower IOP while the angular width of the defects did not depend on the IOP. [6], [7] In our study, we found a greater width of RNFLD in the PF group, though on multivariate analysis, this was not however a significant association. Similar observations have been reported in a study evaluating disc morphology in eyes with and without PF involvement. [17] It may be speculated that structural changes like RNFL thickness are more dependent on the mechanical factors like loss of support connective tissues at the level of the optic nerve while functional changes like those in visual field are more related to vascular factors like proximity to the CVT or autoregulation. Further studies are warranted which explores the differences in regional structure and their association to functional changes in NTG eyes.

This study observed a difference in vertical disc diameter between two eyes of the same patient. Inter-eye asymmetry is a strong indicator of glaucoma. While most studies have studied asymmetric cup disc ratio, few studies like Blue Mountains study have evaluated the asymmetry in disc parameters including vertical disc diameter. [25] This study found a median value for disc diameter asymmetry of 0.07–0.08 mm in glaucoma patients. They however observed that this asymmetry may not be useful for differentiating patients from normals. This observation has caused a major shift in focus from disc asymmetry to asymmetry in cup disc ratio, which is currently the most important determinant for defining glaucoma. Yet, we believe that disc diameter (determining cup size asymmetry) differences in glaucomatous eyes may actually explain differential extent of involvement of both eyes in the same patient. The importance of disc diameter in predisposing an eye to any particular insult may possibly be determined by anatomical differences responsible for disc asymmetry which may also be responsible for anatomical differences in vascular supply. Further studies probing the asymmetry in disc parameters with anatomical differences in vascular supply in glaucoma patients are required to investigate and confirm this possibility.

One of the limitations of our study was that we cannot extrapolate this observation to high myopic eyes since those with high refractive errors or tilted disc (which are potential confounders) were excluded. Also it is unclear if different disc phenotypes like tilted discs would have different CVT exits and therefore alter the susceptibilities of the optic nerve head. We cannot explain why the CVT exit is seen more into the superonasal quadrant in NTG and whether this phenomenon plays a major role in other types of high pressure glaucoma as well.

In conclusion, a greater displacement of the CVT exit from the centre may suggest a higher risk of involvement the central visual field warranting closer follow up and possibly aggressive treatment in bilateral NTG eyes. Further studies evaluating its role in other types of glaucoma and progression of glaucoma is warranted in the future.

## Supporting Information

Table S1 **Shows repeatability coefficients on 10 consecutive images by both examiners for measuring horizontal and vertical distance of central vessels trunk in the study.**
(DOCX)
