# Supplementary material for: Anatomical Attributes of the Optic Nerve Head in Eyes with Parafoveal Scotoma in Normal Tension Glaucoma
Source: PLoS One. 2014 Mar 3;9(3):e90554. doi: 10.1371/journal.pone.0090554 (PMC3940960; doi:10.1371/journal.pone.0090554)
Supplement: Table S1 — Shows repeatability coefficients on 10 consecutive images by both examiners for measuring horizontal and vertical distance of central vessels trunk in the study. (DOCX) [file pone.0090554.s001.docx]

| Examiner 1 | | |
| --- | --- | --- |
| Image number | Repeatability coefficient for Horizontal CVT distance | Repeatability coefficient for Vertical CVT Distance |
| Image 1 | 0.06 | 0.09 |
| Image 2 | 0.07 | 0.06 |
| Image 3 | 0.08 | 0.09 |
| Image 4 | 0.08 | 0.09 |
| Image5 | 0.12 | 0.08 |
| Image 6 | 0.09 | 0.06 |
| Image 7 | 0.06 | 0.08 |
| Image 8 | 0.05 | 0.08 |
| Image 9 | 0.06 | 0.06 |
| Image 10 | 0.08 | 0.06 |
| Examiner 2 | | |
| Image number | Repeatability coefficient for Horizontal CVT distance | Repeatability coefficient for Vertical CVT Distance |
| Image 1 | 0.09 | 0.10 |
| Image 2 | 0.08 | 0.12 |
| Image 3 | 0.08 | 0.07 |
| Image 4 | 0.05 | 0.08 |
| Image5 | 0.06 | 0.08 |
| Image 6 | 0.12 | 0.07 |
| Image 7 | 0.15 | 0.06 |
| Image 8 | 0.06 | 0.09 |
| Image 9 | 0.08 | 0.07 |
| Image 10 | 0.07 | 0.06 |

Table S1: Repeatability coefficients on 10 consecutive images by both examiners (APR & SM) for measuring horizontal and vertical distance of central vessels trunk in the study.

CVT-Central vessel trunk
